# Influence of Van der Waals Interactions on the Solvation Energies of Adsorbates at Pt‐Based Electrocatalysts

**DOI:** 10.1002/cphc.201900512

**Published:** 2019-08-19

**Authors:** Laura P. Granda‐Marulanda, Santiago Builes, Marc T. M. Koper, Federico Calle‐Vallejo

**Affiliations:** ^1^ Leiden Institute of Chemistry Leiden University, PO Box 9502 2300 RA Leiden The Netherlands.; ^2^ Departamento de Ingeniería de Procesos Universidad EAFIT, Carrera 49 No 7 sur – 50 050022 Medellín Colombia; ^3^ Departament de Ciència de Materials i Química Física & Institut de Química Teòrica i Computacional (IQTCUB) Universitat de Barcelona Martí i Franquès 1 08028 Barcelona Spain

**Keywords:** adsorbate solvation, density functional theory, near-surface alloys, oxygen reduction reaction, platinum

## Abstract

Solvation can significantly modify the adsorption energy of species at surfaces, thereby influencing the performance of electrocatalysts and liquid‐phase catalysts. Thus, it is important to understand adsorbate solvation at the nanoscale. Here we evaluate the effect of van der Waals (vdW) interactions described by different approaches on the solvation energy of *OH adsorbed on near‐surface alloys (NSAs) of Pt. Our results show that the studied functionals can be divided into two groups, each with rather similar average *OH solvation energies: (1) PBE and PW91; and (2) vdW functionals, RPBE, PBE‐D3 and RPBE‐D3. On average, *OH solvation energies are less negative by ∼0.14 eV in group (2) compared to (1), and the values for a given alloy can be extrapolated from one functional to another within the same group. Depending on the desired level of accuracy, these concrete observations and our tabulated values can be used to rapidly incorporate solvation into models for electrocatalysis and liquid‐phase catalysis.

## Graphical abstract

**Water solvation** modifies the adsorption energies of *OH, and therefore, also the oxygen‐reduction activity of Pt electrocatalysts. The authors show that *OH solvation on Pt(111) near‐surface alloys depends on the exchange‐correlation functional used and whether long‐range interactions are included or not. They divide the functionals into two groups so that extrapolating *OH solvation energies within a given group is safe. However, extrapolations between different groups should be avoided if accurate activity predictions are to be made.

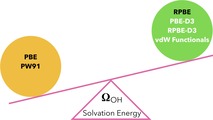

The solvation of adsorbates is becoming a topic of great interest in computational electrocatalysis in view of the current search for more realistic representations of electrode‐electrolyte interfaces. Numerous recent experimental works show that solvent and/or electrolyte effects change the activity and selectivity of electrocatalysts for important reactions such as oxygen reduction,^1^, ^2^ hydrogen evolution,^3^, ^4^ CO_2_ reduction,^5^, ^6^ and CO reduction.^7^, ^8^ In addition, computational works show that solvation and/or cation coadsorption modify the adsorption energies of reaction intermediates,^9^, ^10^, ^11^, ^12^, ^13^ which may not only lead to changes in reaction pathways^14^ but also to considerable differences in the calculated activity of electrocatalysts.^10^, ^15^

In computational electrocatalysis adsorbate‐solvent and adsorbate‐electrolyte interactions at the interface can be evaluated implicitly (where the solvent is modelled as a continuum with certain dielectric constant),^13^, ^16^, ^17^, ^18^, ^19^, ^20^, ^21^, ^22^, ^23^, ^24^, ^25^ explicitly,^26^, ^27^, ^28^, ^29^, ^30^, ^31^, ^32^, ^33^ or through combinations of the two.^34^, ^35^ Furthermore, efforts have been devoted to determine the minimal number of explicit water molecules needed to stabilize a given adsorbate.^36^, ^37^ Such “micro‐solvation” approaches are specifically devised to save computational resources and speedup electrocatalysis modelling. More insight into electrochemical interfaces can be found in ref. [38] and references therein.

The role of vdW forces on water clustering, water‐metal interactions, and liquid water has been explored by using functionals that account for vdW interactions.^39^, ^40^, ^41^, ^42^, ^43^ In general, dispersion‐corrected functionals or those incorporating vdW forces self‐consistently tend to increase the adsorption energy of water on metal surfaces. However, the majority of computational electrocatalysis studies carried out within the framework of Density Functional theory (DFT) use common exchange‐correlation (xc) functionals that do not account for van der Waals (vdW) interactions. Importantly, those might be necessary for an accurate description of water‐water, water‐electrode and water‐adsorbate interactions.

Therefore, here we study the role of vdW interactions on the solvation energy of hydroxyl (*OH) adsorbed on near‐surface alloys (NSAs) of Pt and late transition metals. Those versatile alloys are salient model catalysts for a variety of electrocatalytic reactions.^44^, ^45^, ^46^, ^47^ We observe that: (i) the predictions of *OH solvation at Pt NSAs are comparable for PBE and PW91 (group 1); (ii) The predictions of *OH solvation at Pt NSAs are comparable among RPBE, vdW functionals and GGAs with dispersion corrections (group 2); and (iii) The *OH solvation energies decrease on average by ∼0.14 eV from functionals in group 2 with respect to those in group 1.

## Computational Details

**Figure 1:**
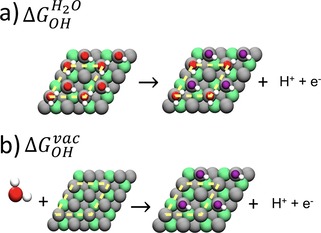
Schematics of Equations 1 and 2 for the free energies of formation of *OH on Pt NSAs. (a) 1/3 ML *OH coadsorbed with 1/3 ML *H_2_O (right side) using as a reference a water bilayer (left side) with one water molecule parallel to the surface plane and the other one with a hydrogen atom pointing towards the surface. (b) 1/3 ML *OH in vacuum (right side) using liquid water as a reference (left side). In both cases the slabs are 2×2 repetitions of a (111) √3×√3 R30° supercell defined by the yellow dashed lines. Each layer in the slab contains 3 metal atoms. Pt: gray, subsurface metal: green, O in H_2_O: red, O in OH: purple, H: white. Top and side views of the water adlayer are shown in Figure S3.

DFT calculations were carried out using the PAW^48^ method in the Vienna Ab initio Simulation Package.^49^ We simulated √3×√3 R30° slabs of Pt(111) NSAs with 1 monolayer (ML) of late transition metal atoms in the subsurface (see Figure 1) and: (I) 1/3 ML *OH in vacuum, (II) 1/3 ML *OH+1/3 ML *H_2_O, and (III) 2/3 ML *H_2_O. The subsurface metals were Co, Ni, Cu, Rh, Pd, Ag, Ir, Pt and Au. The slabs contained four atomic layers: the two uppermost layers and the adsorbates were fully relaxed, while the two bottommost layers were fixed at the converged interatomic distances of bulk Pt calculated for each exchange‐correlation functional. All of the calculated lattice constants appear in Table S8 in the SI. H_2_ and H_2_O were simulated in cubic boxes of 3375 Å^3^. In all simulations, we used 0.01 eV Å^−1^ as convergence criterion for the maximal forces on the atoms, and a plane‐wave cutoff of 450 eV. The k‐point samplings were 8×8×1 for the slabs and 1×1×1 for H_2_ and H_2_O. For the slabs, ∼15 Å of vacuum and dipole corrections in the z direction were used to avoid artificial electrostatic interactions between periodic images. The Methfessel‐Paxton method^50^ was employed to smear the Fermi level of the slabs with k_B_T=0.2 eV, whereas Gaussian smearing was used for H_2_ and H_2_O with k_B_T=0.001 eV. In both cases, all energies were extrapolated to 0 K. We used the computational hydrogen electrode to assess the energetics of solvated protons and electrons.^51^ As a first approximation, we made spin‐restricted calculations for Ni‐ and Co‐containing NSAs. The free energies were evaluated as: G=E_DFT_+ZPE‐TS. The zero‐point energy corrections (ZPE) of gases and adsorbates, as well as the adsorbates’ vibrational entropies were calculated within the harmonic‐oscillator approximation. The ${TS}_{total}^{298.15K}$ corrections for H_2_(g) and H_2_O(l) are 0.40 and 0.67 eV.^51^ We used the following exchange‐correlation functionals: PW91;^52^ PBE;^53^ RPBE;^54^ functionals with semi‐empirical corrections, PBE‐D3 and RPBE‐D3, using the DFT−D3 method;^55^ a functional that evaluates vdW interactions with an optimized version of vdW‐DF method,^56^ namely optPBE;^43^ and the BEEF‐vdW functional.^57^ Specific energetic and geometric data related to this study appear in the Supporting Information, Figure S3 and Tables S1–S8.

## Results and Discussion

Following previous works,^11^, ^37^ to obtain the free energy of solvation (Ω_*OH*_) for *OH on the √3×√3 R30° slab, we determined: (i) the free energy of formation of 1/3 ML *OH coadsorbed with 1/3 ML *H_2_O with respect to an ice‐like water bilayer with 2/3 ML *H_2_O ($\Delta G_{OH}^{H_{2}O}$ ),^41^, ^58^, ^59^ as shown in Equation (1) and in Figure 1a; and (ii) The free energy of formation of 1/3 ML *OH in vacuum with respect to H_2_O(l) ($\Delta G_{OH}^{vac}$ ) using Equation (2), as depicted in Figure 1b.

(1) $$2{}^{*}H{}_{2}O\to {}^{*}\mathrm{OH}+{}^{*}H{}_{2}O+H{}^{+}+e{}^{-}$$

(2) $${}^{*}+H{}_{2}O\left(l\right)\to {}^{*}\mathrm{OH}+H{}^{+}+e{}^{-}$$

where * represents an active site at the NSAs surface. Recent works have shown that three *H_2_O molecules are required to solvate *OH, as the O atom in *OH can make two hydrogen bonds with surrounding water molecules and the H atom can make one.^37^ This is also the case for the periodic water bilayers considered here, as shown in Figure 1a. The difference between Equations (1) and (2) gives the solvation contribution to the free energy of adsorption (Ω_*OH*_):

(3) $$\Omega {}_{OH}=\Delta G_{OH}^{H_{2}O}-\Delta G_{OH}^{vac}$$

Once Ω_*OH*_ is known, it can be added to other adsorption energies calculated in vacuum $\left(\Delta G_{OH}^{vac\#}\right)$ to obtain a first assessment of the adsorption energies in solution ($\Delta G_{OH}^{H_{2}O\#}$ ), which are usually burdensome to calculate:^37^

(4) $$\Delta G_{OH}^{H_{2}O\#}\approx \Delta G_{OH}^{vac\#}+\Omega_{OH}$$

where the superscript # indicates an extrapolation, hence the approximate sign. Previous works concluded that one can extrapolate Ω_*OH*_ from one facet to another of a given material,^37^ but that it is not advisable to do among different materials.^9^, ^10^, ^11^ In the following, we will analyze whether such extrapolations are possible when Ω_*OH*_ is calculated with a different functional than $\Delta G_{OH}^{vac}$ . This is a common situation when using tabulated results from previous works.

Table 1 provides the solvation corrections obtained for *OH with different functionals for nine different Pt NSAs. We have split the functionals in two groups: group 1, formed by PW91 and PBE, and group 2, formed by RPBE, PBE‐D3, RPBE‐D3, optPBE and BEEF‐vdw. In the following, we explain the creation of two groups and the presence of RPBE (a GGA) in group 2 (vdW and dispersion‐corrected functionals) instead of group 1 (other GGAs).

**Table 1:** Free energies of solvation (Ω_*OH*_) in eV for 1/3 ML *OH coadsorbed with 1/3 ML *H_2_O within a water bilayer using different functionals. avg1 and avg2 are the averages of the solvation energies for group 1 functionals (PBE and PW91) and group 2 functionals (RPBE, vdW and with dispersion corrections) across the same metal. Stdev1/stdev2 are the corresponding standard deviations of avg1/avg2. MAX and MIN are the maximal and minimal values in the dataset across the same functional. Range is the difference between MAX and MIN.

metal	PW91	PBE	RPBE	PBE‐D3	RPBE‐D3	optPBE	BEEF‐vdw	avg1	avg2	stdev1	stdev2
Co	−0.60	−0.69	−0.50	−0.45	–	−0.52	−0.48	−0.64	−0.49	0.07	0.03
Rh	−0.61	−0.61	−0.45	−0.47	−0.48	−0.47	−0.39	−0.61	−0.45	0.00	0.04
Ir	−0.63	−0.63	−0.43	−0.50	−0.50	−0.49	−0.43	−0.63	−0.47	0.00	0.04
Ni	−0.53	−0.52	−0.40	−0.43	−0.45	−0.43	−0.44	−0.53	−0.43	0.01	0.02
Pd	−0.56	−0.56	−0.36	−0.40	−0.41	−0.40	−0.39	−0.56	−0.39	0.00	0.02
Pt	−0.62	−0.62	−0.50	−0.45	−0.57	−0.45	−0.44	−0.62	−0.48	0.00	0.05
Cu	−0.50	−0.42	−0.32	−0.31	−0.27	−0.29	−0.28	−0.46	−0.29	0.06	0.02
Ag	−0.40	−0.38	−0.26	−0.25	−0.27	−0.25	−0.27	−0.39	−0.26	0.02	0.01
Au	−0.49	−0.50	−0.35	−0.35	−0.46	−0.35	−0.33	−0.49	−0.37	0.01	0.05
mean	−0.55	−0.55	−0.39	−0.40	−0.43	−0.41	−0.38				
											
stdev	0.08	0.10	0.08	0.08	0.11	0.09	0.07				
MAX	−0.40	−0.38	−0.26	−0.25	−0.27	−0.25	−0.27				
MIN	−0.63	−0.69	−0.50	−0.50	−0.57	−0.52	−0.48				
range	0.23	0.32	0.24	0.25	0.30	0.27	0.21

Although PBE and PW91 are not entirely equivalent,^65^ they do provide similar values for properties such as atomization energies and lattice constants. Indeed, for Pt using PW91 we calculated a lattice constant of 3.99 Å and of 3.98 Å using PBE (see Table S8). Therefore, for adsorption free energies it is expected that the results do not differ significantly. Indeed, we found for Ω_*OH*_ that the average values for PBE/PW91 are similar (see Table 1).

In general, RPBE and PBE/PW91 give different results for properties such as atomization energies for molecules and equilibrium cell volumes for solids.^60^ It has been shown that PBE/PW91 over‐bind adsorbates to surfaces with respect to experiments, while RPBE under‐binds them,^61^ which is presumably connected to RPBE's severe underestimation of surface energies.^62^

Here we observe something similar: the *OH solvation corrections for NSAs, which are the difference between the adsorption energies of *OH in solution and in vacuum $\left(\Omega_{OH}=\Delta G_{OH}^{H_{2}O}-\Delta G_{OH}^{vac}\right)$ , are in average ∼0.15 eV more negative for PBE compared to RPBE (see Table 1). This is because on average $\Delta G_{OH}^{vac}$ is 0.11 eV weaker for RPBE vs PBE, whereas $\Delta G_{OH}^{H_{2}O}$ is weaker by 0.26 eV (see Tables S2 and S3). We note that the trends in Ω_*OH*_, $\Delta G_{OH}^{H_{2}O}$ , and $\Delta G_{OH}^{vac}$ as a function of the number of valence electrons (see Table 1 and Figure S2) are similar for PBE, PW91, and RPBE.

The less negative average solvation energy with respect to PBE/PW91 is also observed for functionals incorporating vdW interactions and dispersion corrections. This is because the adsorbate‐metal interactions are more strongly enhanced for $\Delta G_{OH}^{vac}$ compared to $\Delta G_{OH}^{H_{2}O}$ , in view of the presence of H_2_O in the latter.^39^ On average, $\Delta G_{OH}^{vac}$ is strengthened by 0.17 eV for PBE‐D3 with respect to PBE, whereas $\Delta G_{OH}^{H_{2}O}$ is strengthened only by 0.02 eV (see Tables S2 and S3). We attribute this to the enhanced water‐substrate interactions provided by dispersion corrections: the average adsorption energy of the water bilayer is made more negative by 0.22 eV for PBE‐D3 with respect to PBE (see Table S4). Indeed, it is well known that dispersion corrections increase the binding energy of water adlayers on substrates,^43^ which according to Equations (1) and (3) makes the solvation energies less negative.

Altogether, in group 1 we have PBE and PW91, which are over‐binding GGAs, while in group 2 we have under‐binding GGAs such as RPBE together with vdW functionals and dispersion‐corrected functionals. The average solvation energy for the functionals in group 1 is −0.55 eV, while it is −0.40 eV for the functionals in group 2. We emphasize that the division of functionals in groups 1 and 2 is based merely on the results, and a rigorous classification would require data from additional functionals and adsorbates.

Regarding the safe use of Equation (4), there are two important points to be considered: first, for a given functional, the variations among the different alloys are large. The standard deviation for the alloys in groups 1 and 2 is 0.09 and 0.08 eV, respectively. In line with previous works focused on PBE only,^11^ our conclusion is that it is unadvisable to use a single solvation correction for all alloys. Second, for a given alloy one can combine among functionals from either group 1 or group 2, but not between groups. The means of the standard deviations for the alloys are 0.03 and 0.02 eV for groups 1 and 2, respectively. Essentially, applying PBE‐calculated solvation energies to PW91’s adsorption energies in vacuum (and vice versa) is possible using Equation (4), as the solvation corrections are comparable for both functionals. Ω_*OH*_ values can also be extrapolated from one functional to another within group 2. However, it is preferable not to extrapolate values of group 1 to group 2 and vice versa, as the differences are on average ∼0.15 eV. For instance, the design principle for oxygen reduction is that optimal catalysts should bind around 0.10–0.15 eV weaker than Pt(111) ($\Delta G_{OH}-\Delta G_{OH}^{Pt(111)}\approx 0.1-0.15\;eV$ ),^11^, ^63^, ^64^ so it is advisable to avoid intergroup extrapolations. Table 1 provides the average values (avg1/avg2) and standard deviations (stdev1/stdev2) for each alloy in each group. Figure S2 shows the energy trends for the two groups. The average and standard deviation for a given alloy across all functionals appear in Table S1.

**Figure 2:**
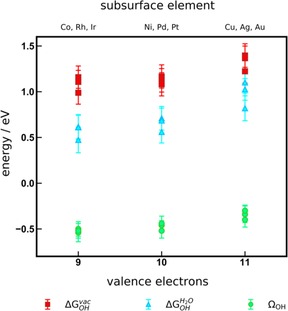
Adsorption energies of 1/3 ML *OH in vacuum (red, $\Delta G_{OH}^{vac}$ ), within the water bilayer (blue, $\Delta G_{OH}^{H_{2}O}$ ), and in the solvation energy (green, Ω_*OH*_), as a function of the number of valence electrons of the subsurface metal atom in the Pt NSAs. The error bars cover the energy range spanned by the different functionals analyzed. The correlation between the number of valence electrons and the d‐band centers of the Pt skins is provided in Figure S1.

Figure 2 summarizes the trends in adsorption energies in vacuum and in solution together with the *OH solvation energies, as a function of the number of valence electrons of the metal in the Pt NSAs (see also Figure S1). The data points are the average values for each NSA considering all functionals (avg0) in Tables S2, S3, and S1 for $\Delta G_{OH}^{vac}$ , $\Delta G_{OH}^{H_{2}O}$ , and Ω_*OH*_ respectively and the error bars correspond to the standard deviation across the metals (stdev0) in Tables S2, S3, and S1, respectively. Importantly, the size of the error bars decreases for Ω_*OH*_ with respect to $\Delta G_{OH}^{vac}$ and $\Delta G_{OH}^{H_{2}O}$ . This shows that: (i) the number of valence electrons of the components can be used to predict solvation corrections, in line with previous works on adsorption‐energy trends;^11^, ^66^, ^67^, ^68^ and (ii) because Ω_*OH*_ results from the difference of $\Delta G_{OH}^{vac}$ and $\Delta G_{OH}^{H_{2}O}$ (Equation 3), its actual values are considerably less functional‐dependent than those of the original adsorption energies.

## Conclusions

Using *OH adsorbed on Pt near‐surface alloys with transition metals as a case study, we showed that accounting for long‐range interactions generally results in a decrease of the strength of solvation contributions to the adsorption energies with respect to GGAs. The decrease is on average ∼0.14 eV and is due to the enhancement of water‐metal interactions when including long‐range interactions. The solvation energies of *OH calculated with PBE are similar to those of PW91 but differ ∼0.15 eV from those of RPBE. Solvation energies calculated with RPBE, vdW functionals and dispersion‐corrected GGA functionals are generally similar. Furthermore, solvation corrections can be predicted based on the number of valence electrons of the subsurface metal in the alloy. Depending on the desired level of accuracy, these guidelines can be used to decide whether specific solvation energies need to be calculated or if average values suffice, which can help in making more efficient electrocatalysis and liquid‐phase catalysis models.

## Conflict of interest

The authors declare no conflict of interest.

## Supporting information

As a service to our authors and readers, this journal provides supporting information supplied by the authors. Such materials are peer reviewed and may be re‐organized for online delivery, but are not copy‐edited or typeset. Technical support issues arising from supporting information (other than missing files) should be addressed to the authors.

Supplementary
