# Supplementary material for: Influence of Van der Waals Interactions on the Solvation Energies of Adsorbates at Pt‐Based Electrocatalysts
Source: Chemphyschem. 2019 Aug 19;20(22):2968–72. doi: 10.1002/cphc.201900512 (PMC6899950; doi:10.1002/cphc.201900512)
Supplement: Supplementary file 1 — Supplementary [file CPHC-20-2968-s001.pdf]

CHEMPHYSCHEM

Supporting Information

© Copyright Wiley-VCH Verlag GmbH & Co. KGaA, 69451 Weinheim, 2019

# Influence of Van der Waals Interactions on the Solvation Energies of Adsorbates at Pt-Based Electrocatalysts

Laura P. Granda-Marulanda, Santiago Builes, Marc T. M. Koper, and Federico Calle-Vallejo*© 2019 The Authors. Published by Wiley-VCH Verlag GmbH & Co. KGaA. This is an open access article under the terms of the Creative Commons Attribution License, which permits use, distribution and reproduction in any medium, provided the original work is properly cited.An invited contribution to a Special Issue on Electrocatalysis

# Supporting Information for:

# Influence of Van der Waals interactions on the solvation energies of adsorbates at Pt-based electrocatalysts

Laura P. Granda-Marulanda,[1] Santiago Builes,[2] Marc T. M. Koper,[1] Federico Calle-Vallejo[3,*]

[1] Leiden Institute of Chemistry, Leiden University, PO Box 9502, 2300 RA Leiden, The Netherlands.

[2] Departamento de Ingeniería de Procesos, Universidad EAFIT, Carrera 49 No 7 sur-50, 050022, Medellín, Colombia.

[3] Departament de Ciència de Materials i Química Física & Institut de Química Teòrica i Computacional (IQTCUB), Universitat de Barcelona, Martí i Franqués 1, 08028 Barcelona, Spain.

* Corresponding author: f.calle.vallejo@ub.edu

In Figure S1 we show that the PBE-calculated[1] d-band center of the Pt skin on the near-surface alloys studied here linearly correlates with the number of valence electrons of the metals along the 3d, 4d and 5d series. It was previously shown that valence electrons correlate linearly with the d-band centers of various transition metals.[1,2]

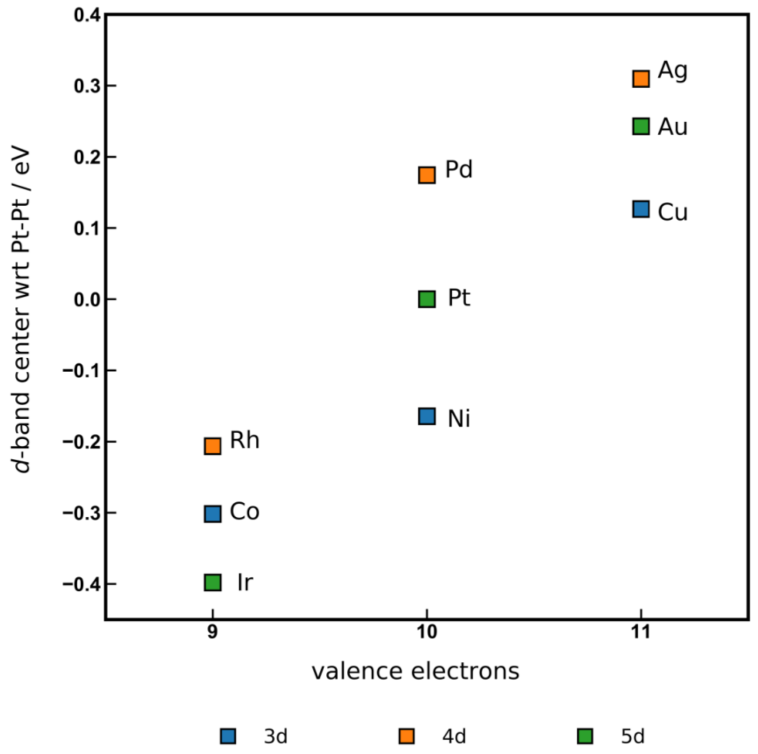


**Figure S1.** D-band center of the Pt skin on the NSAs with respect to the d-band center of the top layer of Pt(111) as a function of the valence electrons of the subsurface metals. A nearly linear correlation between the d-band center and the valence electrons for various metals along the 3d (Co, Ni, Cu), 4d (Rh, Pd, Ag), and 5d (Ir, Pt, Au) series is observed, in line with previous reports.[1,3]

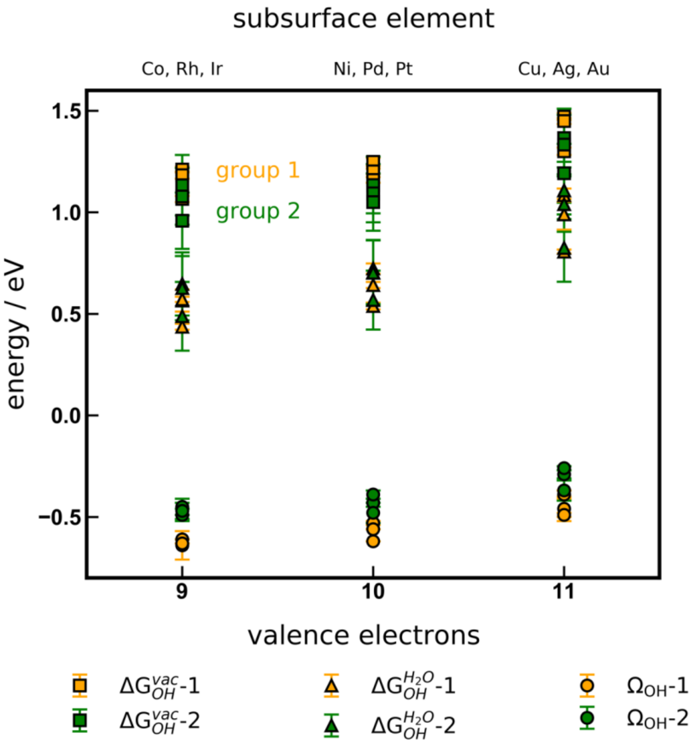


**Figure S2.** Adsorption energies of group 1 functionals (PBE/ PW91) in orange and group 2 functionals (RPBE, vdW and with dispersion corrections) in green as a function of the number of valence electrons of the subsurface metal atom in the Pt NSAs. For both groups, squares represent the energies of 1/3 ML *OH in vacuum ( $\Delta G_{OH}^{vac}$ ) triangles represent the energies of *OH within the water bilayer ( $\Delta G_{OH}^{H_2O}$ ), and circles represent the solvation energy ( $\Omega_{OH}$ ). Solvation energies for group 2 (green) are generally less negative than those of group 1 (orange). The error bars cover the standard deviation of the respective groups of functionals. The correlation between the number of valence electrons and the d-band centers of the Pt skins is provided in Figure S1.

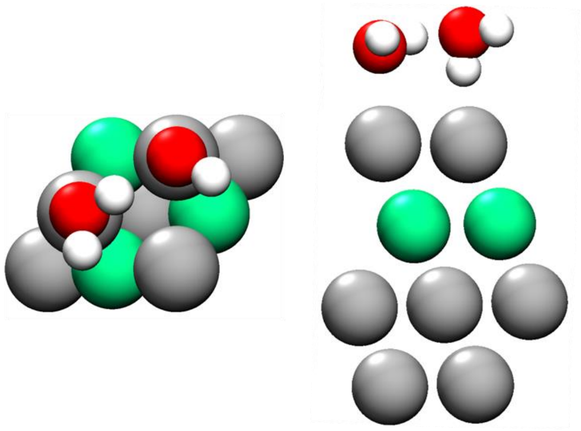

**Figure R1.** Top and side views of the water adlayer on a √3×√3 R30º unit cell of Pt(111) NSA. The cell contains 3 metal atoms per layer, and 2 adsorbed water molecules per unit cell. The water molecules are each on top of a Pt atom. One water molecule is parallel to the surface plane and the other one with one of its hydrogen atoms pointing towards the surface, as shown in the side view.

The water adlayer shown in Figure S3 is the ice-like water adlayer structure found to be computationally stable in closed-packed metal surfaces.[4–6] This water adlayer of 2/3 ML coverage is adsorbed with one water molecule in parallel to the surface plane while the other water molecule adsorbs with one of its hydrogen atoms pointing towards the surface (H-down) or away from it (H-up). The energetic difference between these two configurations is small (~0.05 eV), as reported in the literature based on DFT adsorption energies.[4,7] Our calculated free energies of adsorption of the water adlayer on Pt111 show a difference of 0.01 eV between the H-up and H-down configuration, being the H-down configuration more stable.

**Table S1.** Free energies of solvation ( $\Omega_{OH}$ ) in eV for 1/3 ML *OH coadsorbed with 1/3 ML *$H_2O$ within a water bilayer using different functionals. Avg1 and avg2 are the averages of the solvation energies for group 1 functionals (PBE, PW91) and group 2 functionals (RPBE, vdW and dispersion corrections) across the same metal. Stdev1/2 are the corresponding standard deviations of avg1/avg2. Avg0 is the average of the solvation energies across the metals for all the functionals and stdev0 is its standard deviation. MAX and MIN are the maximal and minimal values in the dataset across the same functional. Range is the difference between MAX and MIN. [a]

| metal | PW91 | PBE | RPBE | PBE-D3 | RPBE -D3 | Opt PBE | BEEF -vdw | avg0 | avg1 | avg2 | stdev0 | stdev1 | stdev2 |
|---|---|---|---|---|---|---|---|---|---|---|---|---|---|
| Co | -0.60 | -0.69 | -0.50 | -0.45 | - | -0.52 | -0.48 | -0.54 | -0.64 | -0.49 | 0.09 | 0.07 | 0.03 |
| Rh | -0.61 | -0.61 | -0.45 | -0.47 | -0.48 | -0.47 | -0.39 | -0.50 | -0.61 | -0.45 | 0.08 | 0.00 | 0.04 |
| Ir | -0.63 | -0.63 | -0.43 | -0.50 | -0.50 | -0.49 | -0.43 | -0.52 | -0.63 | -0.47 | 0.08 | 0.00 | 0.04 |
| Ni | -0.53 | -0.52 | -0.40 | -0.43 | -0.45 | -0.43 | -0.44 | -0.46 | -0.53 | -0.43 | 0.05 | 0.01 | 0.02 |
| Pd | -0.56 | -0.56 | -0.36 | -0.40 | -0.41 | -0.40 | -0.39 | -0.44 | -0.56 | -0.39 | 0.08 | 0.00 | 0.02 |
| Pt | -0.62 | -0.62 | -0.50 | -0.45 | -0.57 | -0.45 | -0.44 | -0.52 | -0.62 | -0.48 | 0.08 | 0.00 | 0.05 |
| Cu | -0.50 | -0.42 | -0.32 | -0.31 | -0.27 | -0.29 | -0.28 | -0.34 | -0.46 | -0.29 | 0.09 | 0.06 | 0.02 |
| Ag | -0.40 | -0.38 | -0.26 | -0.25 | -0.27 | -0.25 | -0.27 | -0.30 | -0.39 | -0.26 | 0.06 | 0.02 | 0.01 |
| Au | -0.49 | -0.50 | -0.35 | -0.35 | -0.46 | -0.35 | -0.33 | -0.40 | -0.49 | -0.37 | 0.08 | 0.01 | 0.05 |
| mean | -0.55 | -0.55 | -0.39 | -0.40 | -0.43 | -0.41 | -0.38 | | | | | | |
| stdev | 0.08 | 0.10 | 0.08 | 0.08 | 0.11 | 0.09 | 0.07 | | | | | | |
| MAX | -0.40 | -0.38 | -0.26 | -0.25 | -0.27 | -0.25 | -0.27 | | | | | | |
| MIN | -0.63 | -0.69 | -0.50 | -0.50 | -0.57 | -0.52 | -0.48 | | | | | | |
| range | 0.23 | 0.32 | 0.24 | 0.25 | 0.30 | 0.27 | 0.21 | | | | | | |
| LNDm | -0.08 | -0.15 | -0.10 | -0.10 | -0.14 | -0.11 | -0.10 | | | | | | |
| LPDm | 0.15 | 0.17 | 0.14 | 0.16 | 0.16 | 0.16 | 0.11 | | | | | | |
| LPDPt | 0.22 | 0.24 | 0.24 | 0.21 | 0.30 | 0.21 | 0.17 | | | | | | |
| LNDPt | -0.01 | -0.07 | 0.00 | -0.04 | 0.00 | -0.06 | -0.04 | | | | | | |
| AOM | | | | | | -0.44 | | | | | | | |
| Stdev AOM | | | | | | 0.07 | | | | | | | |
| $LND_{AOM}$ | | | | | | -0.10 | | | | | | | |
| $LPD_{AOM}$ | | | | | | 0.06 | | | | | | | |
| avg1 across functionals | | | | | | -0.55 | | | | | | | |
| avg2 across functionals | | | | | | -0.40 | | | | | | | |
| Diff avg1 - avg2 across functionals | | | | | | 0.15 | | | | | | | |
| Diff avg1 - avg2 across metals | | | | | | 0.14 | | | | | | | |

[a] LNDm/LPDm: Largest negative/positive deviation from the mean
LNDPt/LPDPt: Largest negative/positive deviation from Pt
AOM: Average of the means
Standard deviation of AOM
$LND_{AOM}$/$LPD_{AOM}$: Largest negative/positive deviation from the AOM

**Table S2.** Adsorption energies in eV of 1/3 ML *OH in vacuum ( $\Delta G_{OH}^{vac}$ ). avg1 and avg2 are the averages of the solvation energies for group 1 functionals (PBE, PW91) and group 2 functionals (RPBE, vdW and with dispersion corrections) across the same metal. Stdev1/2 are the corresponding standard deviations of avg1/avg2. Avg0 is the average of the solvation energies across the metals for all the functionals and stdev0 is its standard deviation.

| metal | PW91 | PBE | RPBE | PBE-D3 | RPBE-D3 | optPBE | BEEF-vdw | avg0 | avg1 | avg2 | stdev0 | stdev1 | stdev2 |
|---|---|---|---|---|---|---|---|---|---|---|---|---|---|
| Co | 1.20 | 1.22 | 1.33 | 1.11 | - | 0.97 | 1.12 | 1.16 | 1.21 | 1.13 | 0.12 | 0.01 | 0.15 |
| Rh | 1.17 | 1.19 | 1.31 | 1.03 | 1.02 | 0.94 | 1.10 | 1.11 | 1.18 | 1.08 | 0.13 | 0.02 | 0.14 |
| Ir | 1.05 | 1.08 | 1.19 | 0.91 | 0.89 | 0.82 | 0.98 | 0.99 | 1.07 | 0.96 | 0.12 | 0.02 | 0.14 |
| Ni | 1.23 | 1.26 | 1.36 | 1.08 | 1.08 | 0.99 | 1.15 | 1.17 | 1.25 | 1.13 | 0.13 | 0.02 | 0.14 |
| Pd | 1.19 | 1.21 | 1.33 | 1.04 | 1.04 | 0.96 | 1.11 | 1.12 | 1.20 | 1.09 | 0.13 | 0.01 | 0.14 |
| Pt | 1.15 | 1.17 | 1.28 | 0.99 | 0.99 | 0.91 | 1.07 | 1.08 | 1.16 | 1.05 | 0.13 | 0.01 | 0.14 |
| Cu | 1.44 | 1.46 | 1.57 | 1.28 | 1.24 | 1.20 | 1.37 | 1.37 | 1.45 | 1.33 | 0.13 | 0.01 | 0.15 |
| Ag | 1.46 | 1.48 | 1.60 | 1.31 | 1.29 | 1.23 | 1.40 | 1.40 | 1.47 | 1.37 | 0.13 | 0.02 | 0.14 |
| Au | 1.29 | 1.31 | 1.43 | 1.14 | 1.13 | 1.05 | 1.22 | 1.22 | 1.30 | 1.19 | 0.13 | 0.02 | 0.15 |

**Table S3.** Adsorption energies in eV of 1/3 ML *OH coadsorbed with 1/3ML *H2O ( $\Delta G_{OH}^{H_2O}$ ). Avg1 and avg2 are the averages of the solvation energies for group 1 functionals (PBE, PW91) and group 2 functionals (RPBE, vdW and with dispersion corrections) across the same metal. Stdev1/2 are the corresponding standard deviations of avg1/avg2. Avg0 is the average of the solvation energies across the metals for all the functionals and stdev0 is its standard deviation.

| metal | PW91 | PBE | RPBE | PBE-D3 | RPBE-D3 | optPBE | BEEF-vdw | avg0 | avg1 | avg2 | stdev0 | stdev1 | stdev2 |
|---|---|---|---|---|---|---|---|---|---|---|---|---|---|
| Co | 0.60 | 0.53 | 0.83 | 0.66 | - | 0.45 | 0.64 | 0.62 | 0.57 | 0.65 | 0.13 | 0.05 | 0.16 |
| Rh | 0.56 | 0.58 | 0.86 | 0.55 | 0.54 | 0.47 | 0.72 | 0.61 | 0.57 | 0.63 | 0.13 | 0.01 | 0.16 |
| Ir | 0.43 | 0.45 | 0.76 | 0.41 | 0.39 | 0.34 | 0.55 | 0.47 | 0.44 | 0.49 | 0.14 | 0.01 | 0.17 |
| Ni | 0.70 | 0.74 | 0.97 | 0.65 | 0.63 | 0.56 | 0.72 | 0.71 | 0.72 | 0.70 | 0.13 | 0.03 | 0.16 |
| Pd | 0.63 | 0.65 | 0.97 | 0.64 | 0.63 | 0.55 | 0.73 | 0.69 | 0.64 | 0.70 | 0.14 | 0.02 | 0.16 |
| Pt | 0.53 | 0.55 | 0.78 | 0.54 | 0.43 | 0.46 | 0.63 | 0.56 | 0.54 | 0.57 | 0.12 | 0.02 | 0.15 |
| Cu | 0.94 | 1.04 | 1.25 | 0.97 | 0.97 | 0.91 | 1.09 | 1.03 | 0.99 | 1.04 | 0.12 | 0.08 | 0.14 |
| Ag | 1.06 | 1.11 | 1.34 | 1.07 | 1.03 | 0.98 | 1.13 | 1.10 | 1.08 | 1.11 | 0.12 | 0.03 | 0.14 |
| Au | 0.80 | 0.81 | 1.08 | 0.79 | 0.66 | 0.70 | 0.89 | 0.82 | 0.81 | 0.82 | 0.14 | 0.01 | 0.17 |

**Table S4.** Normalized adsorption energies in eV/$H_2O$ molecule of 2/3 ML water adlayer on the Pt NSAs for the different functionals studied ( $2*+2H_2O(l) \rightarrow 2*H_2O$ , $\Delta G_{H_2O}$ ).

| metal | PW91 | PBE | RPBE | PBE-D3 | RPBE-D3 | optPBE | BEEF-vdw |
|---|---|---|---|---|---|---|---|
| Co | 0.07 | 0.15 | 0.24 | -0.09 | - | -0.01 | 0.11 |
| Rh | 0.10 | 0.12 | 0.23 | -0.10 | -0.01 | -0.02 | 0.08 |
| Ir | 0.10 | 0.12 | 0.21 | -0.10 | -0.01 | -0.02 | 0.09 |
| Ni | 0.08 | 0.10 | 0.23 | -0.09 | 0.00 | -0.01 | 0.13 |
| Pd | 0.10 | 0.13 | 0.21 | -0.10 | -0.02 | -0.03 | 0.11 |
| Pt | 0.10 | 0.13 | 0.24 | -0.11 | -0.02 | -0.03 | 0.11 |
| Ag | 0.09 | 0.10 | 0.22 | -0.12 | -0.02 | -0.04 | 0.10 |
| Au | 0.09 | 0.12 | 0.23 | -0.11 | -0.02 | -0.03 | 0.10 |
| Cu | 0.12 | 0.11 | 0.23 | -0.10 | -0.03 | -0.03 | 0.09 |

**Table S5.** Distances in Å between the oxygen of the water lying flat within the 2/3 ML water adlayer and the nearest Pt atom for the different functionals studied.

| metal | PW91 | PBE | RPBE | PBE-D3 | RPBE-D3 | optPBE | BEEF-vdw |
|---|---|---|---|---|---|---|---|
| Co | 3.46 | 3.52 | 4.26 | 2.74 | - | 2.78 | 3.84 |
| Rh | 3.03 | 3.05 | 4.38 | 2.69 | 2.77 | 3.00 | 3.49 |
| Ir | 3.00 | 3.03 | 4.53 | 2.62 | 2.72 | 2.99 | 3.49 |
| Ni | 3.43 | 3.47 | 4.36 | 2.71 | 2.77 | 2.85 | 3.71 |
| Pd | 2.88 | 2.91 | 4.64 | 2.67 | 2.75 | 2.98 | 3.29 |
| Pt | 2.82 | 2.89 | 4.73 | 2.66 | 2.74 | 2.96 | 3.26 |
| Ag | 3.05 | 3.10 | 4.49 | 2.91 | 2.89 | 3.06 | 3.27 |
| Au | 2.97 | 3.02 | 4.46 | 2.79 | 2.81 | 2.98 | 3.21 |
| Cu | 3.31 | 3.32 | 4.46 | 2.87 | 2.85 | 3.13 | 3.74 |

**Table S6.** Distance in Å between the oxygen of the water lying flat in the 1/3 ML *OH coadsorbed with 1/3 ML $H_2O$ and the nearest Pt atom.

| metal | PW91 | PBE | RPBE | PBE-D3 | RPBE-D3 | optPBE | BEEF-vdw |
|---|---|---|---|---|---|---|---|
| Co | 2.27 | 2.27 | 2.32 | 2.25 | - | 2.29 | 2.34 |
| Rh | 2.25 | 2.25 | 2.31 | 2.25 | 2.28 | 2.28 | 2.33 |
| Ir | 2.23 | 2.23 | 2.28 | 2.23 | 2.26 | 2.26 | 2.30 |
| Ni | 2.27 | 2.27 | 2.33 | 2.27 | 2.30 | 2.30 | 2.35 |
| Pd | 2.25 | 2.26 | 2.32 | 2.25 | 2.29 | 2.28 | 2.33 |
| Pt | 2.23 | 2.23 | 2.28 | 2.22 | 2.27 | 2.25 | 2.30 |
| Cu | 2.34 | 2.33 | 2.43 | 2.32 | 2.37 | 2.37 | 2.46 |
| Ag | 2.34 | 2.35 | 2.49 | 2.33 | 2.40 | 2.39 | 2.48 |
| Au | 2.28 | 2.28 | 2.37 | 2.27 | 2.31 | 2.32 | 2.40 |

**Table S7.** Zero point energies (ZPEs) in eV for molecules in the gas phase.

| Functional | $H_2$(g) | $H_2O$ (g) |
|---|---|---|
| PW91 | 0.269 | 0.568 |
| PBE | 0.268 | 0.568 |
| RPBE | 0.270 | 0.568 |
| PBE-D3 | 0.268 | 0.568 |
| RPBE-D3 | 0.270 | 0.567 |
| optPBE | 0.271 | 0.566 |
| BEEF-vdw | 0.277 | 0.577 |

**Table S8.** Optimized lattice constants (a) in Å for bulk Pt.

| Functional | a |
|---|---|
| PW91 | 3.99 |
| PBE | 3.98 |
| RPBE | 4.00 |
| PBE-D3 | 3.93 |
| RPBE-D3 | 3.95 |
| optPBE | 4.00 |
| BEEF-vdw | 4.00 |

**References:**


[1] F. Calle-Vallejo, J. I. Martínez, J. M. García-Lastra, J. Rossmeisl, M. T. M. Koper, *Phys. Rev. Lett.* **2012**, *108*, 116103.

[2] H.-Y. Su, K. Sun, W.-Q. Wang, Z. Zeng, F. Calle-Vallejo, W.-X. Li, *J. Phys. Chem. Lett.* **2016**, *7*, 5302–5306.

[3] Z.-D. He, S. Hanselman, Y.-X. Chen, M. T. M. Koper, F. Calle-Vallejo, *J. Phys. Chem. Lett.* **2017**, *8*, 2243–2246.

[4] S. Schnur, A. Groß, *New J. Phys.* **2009**, *11*, 125003.

[5] H. Ogasawara, B. Brena, D. Nordlund, M. Nyberg, A. Pelmenschikov, L. G. M. Pettersson, A. Nilsson, *Phys. Rev. Lett.* **2002**, *89*, 276102.

[6] A. Michaelides, P. Hu, *J. Am. Chem. Soc.* **2001**, *123*, 4235–4242.

[7] A. Michaelides, A. Alavi, D. A. King, *Phys. Rev. B* **2004**, *69*, 113404.
